# Association of LVV-Hemorphin-7 with Sepsis and Shock: Roles of Cathepsin D and G in Hemoglobin Metabolism in a Prospective ICU Cohort Study

**DOI:** 10.3390/biomedicines12122789

**Published:** 2024-12-09

**Authors:** Yao-Kuang Wu, Hsueh-Wen Chung, Yi-Ting Chen, Hsing-Chun Chen, I-Hung Chen, Wen-Lin Su

**Affiliations:** 1 Division of Pulmonary and Critical Care Medicine, Department of Internal Medicine, Taipei Tzu Chi Hospital, Buddhist Tzu Chi Medical Foundation, New Taipei 231, Taiwan; drbfci@gmail.com; 2 School of Medicine, Tzu Chi University, Hualien 970, Taiwan; kateytc@gmail.com; 3 Department of Nursing, College of Nursing, National Yang Ming Chiao Tung University, Taipei City 112, Taiwan; snow721103@gmail.com; 4 Division of Pulmonary and Critical Care Medicine, Department of Internal Medicine, Hualien Tzu Chi Hospital, Buddhist Tzu Chi Medical Foundation, Hualien 970, Taiwan; 5 Division of Pulmonary and Critical Care Medicine, Department of Internal Medicine, Dalin Tzu Chi Hospital, Buddhist Tzu Chi Medical Foundation, Chiayi County 622, Taiwan; dm689688@tzuchi.com.tw (H.-C.C.); b89401098@ntu.edu.tw (I.-H.C.)

**Keywords:** sepsis, shock, cathepsin, LVV-hemorphin-7, sequential organ failure assessment score

## Abstract

Background: Sepsis is a leading cause of mortality in intensive care units (ICUs). Cell-free hemoglobin (CFH) released during sepsis interacts with lysosomal enzymes from neutrophils and macrophages. This study aims to examine the association of LVV-hemorphin-7 (LVV-H7), cathepsin D, and cathepsin G with sepsis and shock in ICU patients. Methods: A prospective observational cohort study was conducted in the medical ICU of a tertiary referral hospital in Taiwan. The patients with an acute increasing sequential organ failure assessment (SOFA) score ≥ 2 between 2022 and 2023. Blood samples from 40 healthy controls were obtained from the hospital biobank. CFH metabolites, including LVV-H7 and lysosomal enzyme cathepsin D and cathepsin G, were compared between the sepsis (definite and probable) and non-sepsis (possible sepsis) groups. Multivariate logistic regression analyzed factors associated with sepsis and shock. Results: Among 120 patients, 75 were classified as septic and 45 as non-septic. Significant differences were observed in CFH, cathepsin D, cathepsin G, and LVV-H7 levels between sepsis and non-sepsis groups. LVV-H7 was a significant predictor for sepsis (adjusted OR [aOR] 1.009, 95% CI 1.005–1.013; *p* < 0.001) and shock (aOR 1.005, 95% CI 1.002–1.008; *p* < 0.05). Cathepsin G predicted non-shock (aOR 0.917, 95% CI 0.848–0.991; *p* < 0.05), while cathepsin D predicted septic shock (aOR 1.001, 95% CI 1.000–1.002; *p* < 0.05). Conclusions: LVV-H7, cathepsin D, and cathepsin G are associated with the classification of sepsis and shock episodes in critically ill patients with elevated SOFA scores.

## 1. Introduction

Sepsis is an extreme response to an infection that causes significant erythrocyte damage, particularly in intensive care units (ICUs) where patients often develop anemia [1,2,3]. During sepsis-induced anemia, cell-free hemoglobin (CFH) is released by erythrocytes through a series of mechanisms [4]. Traditionally, free heme and CFH-damaged tissue have been observed to worsen sepsis in lipopolysaccharide-induced systemic inflammatory animal models [5,6]. Clinical studies have shown that higher levels of CFH in patients with sepsis are associated with lower survival rates [7,8].

In infection cases, neutrophils and macrophages are recruited to the infection site to combat pathogen invasion. Cathepsin G originates from neutrophils [9,10], while cathepsin D comes from the lysosomes of macrophages [11]. These enzymes are potentially involved in mechanisms that play a critical role in Hb cleavage during sepsis and the production of hemorphins [12]. Hemorphin-7 (H7), which includes LVV-H7 and VV-H7, is defined as an Hb metabolite found in thrombotic tissue and abdominal aortic aneurysms that attracts leukocytes [13]. This suggests a synergistic effect between Hb and immune cells during sepsis and the possible release of LVV-H7 into the blood, attracting leucocyte recruitment. Recent cell culture studies have shown that LVV-H7 inhibits the angiotensin-converting enzyme, downregulating angiotensin II and decreasing blood pressure, suggesting a negative correlation between LVV-H7 levels and shock [14]. The LVV-H7 may have synergism that increases the risk of shock. These findings would justify the need for assessing the hemoglobin metabolism-related biomarkers of sepsis severity.

This study was the first investigation about CFH, lysosomal enzymes, and LVV-H7 in critical sepsis cases and compared these biomarkers between sepsis and control groups to identify potential diagnostic factors for sepsis and shock.

## 2. Methods

### 2.1. Study Design and Participant Enrolment

**Figure 1:**
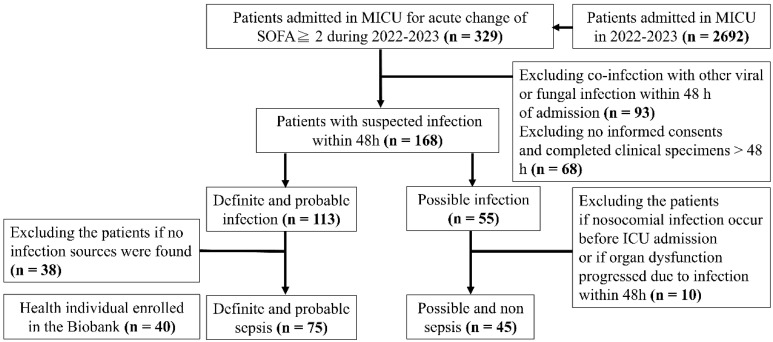
Cohort flow diagram of the sepsis study enrollment process. MICU: Medical intensive care unit; ICU: intensive care unit; SOFA, Sequential Organ Failure Assessment Score.

This prospective observational cohort study consisted of patients with suspected sepsis admitted to the medical intensive care units (ICUs) at a tertiary referral medical center in Taiwan from 2022 to 2023, as shown in Figure 1.

The study protocol titled “Exploring the anti-inflammation mechanisms of hemoglobin and its metabolites in sepsis” was approved by the Institutional Review Board of Taipei Tzu Chi Hospital, Buddhist Tzu Chi Medical Foundation, New Taipei City, Taiwan, on 16 March 2020 (Protocol No.: 08-P-134). To evaluate the Hb metabolite concentrations in the general population, the study titled “Analysis of erythrocyte-related metabolites in plasma of healthy adults using biobank specimens” was approved by the same board on 11 January 2024 (Protocol No.: 13-IRB006). Forty blood samples previously collected from healthy participants were applied as healthy controls. The informed consent was waived, and the Biobank Ethics Committee approved this application on 12 January 2024 (application No: Tzubiobank 2024-05). All study procedures adhered to the ethical standards of the responsible committee on human experimentation and the Helsinki Declaration of 1975. Informed consents were obtained from participants or their legal agents upon ICU admission.

Inclusion criteria included patients meeting the sequential organ failure assessment (SOFA) score ≥ 2 and suspected infection since initial ICU admission. Suspected infections were categorized as definite, probable, or possible based on clinical symptoms and radiological evidence according to the International Sepsis Forum Criteria [15]. The sepsis classifications were divided into definite, probable, and possible sepsis categories following the criteria established by Rhee et al. [16]. and Mellhammar et al. [17], as detailed in the following.

Definite and probable sepsis were defined by the presence of criterion 1 and either criterion 2 or 3:

1. A definite or probable source of infection (e.g., positive cultures or radiographic evidence with a compatible clinical syndrome).
2. Organ dysfunction due to infection without any discernable cause other than infection.
3. Organ dysfunction most likely attributable to infection, although other potential contributors were present.

Possible sepsis was defined by the presence of either criterion 1 or 2:

1. Patients were treated for presumed sepsis but lacked definitive evidence of infection.
2. Patients had alternative definite or possible explanations for organ dysfunction.

In our study, the sepsis group included patients classified as having definite or probable sepsis, while the non-sepsis group included those classified as having possible sepsis.

Exclusion criteria: Patients were excluded if they had co-infections with other viral or fungal pathogens within 48 h of admission or if the collection of clinical specimens was delayed beyond 48 h after admission, as these data would not reflect early sepsis conditions. After applying these exclusion criteria, patients were retrospectively classified into either the sepsis (definite and probable) or non-sepsis (possible sepsis) groups based on the established sepsis classification criteria.

Subgroup exclusion criteria: In the “definite and probable sepsis” group, patients were excluded if no source of microbiologic infection was identified. In the “possible and non-sepsis” group, patients were excluded if nosocomial infection occurred before ICU admission or if organ dysfunction progressed due to infection within 48 h.

The identified pathogens from cultures and the primary sources of infection in the sepsis (definite and probable) group were documented. The causes of organ dysfunction in the non-sepsis (possible sepsis) group were documented and are available in the following seven groups.

1. Acute or chronic renal failure leading to acute pulmonary edema with hypoxemic respiratory failure or hyperkalemia with bradycardic heart failure.
2. Cardiac dysfunction-related cardiogenic hypotension, including acute myocardial infarction, decompensated heart failure, and arrhythmias.
3. Pulmonary disease causing impaired gas exchange and hypoxemia, such as chronic obstructive pulmonary disease, asthma, and interstitial lung disease.
4. Gastrointestinal tract hemorrhage with hypovolemic hypotension, including esophageal varices bleeding and upper or lower gastrointestinal tract bleeding.
5. Acute pancreatitis with volume depletion and distributive hypotension.
6. Metabolic acidosis-related hypotension or cardiac suppression, including diabetic ketoacidosis and hyperglycemic hyperosmolar syndrome.
7. Anaphylaxis resulting in distributive hypotension.

Patients identified as having septic shock were those requiring vasopressors to maintain a mean arterial pressure (MAP) ≥ 65 mmHg [18] due to persistent hypotension with a serum lactate level > 2 mmol/L (18 mg/dL), despite sufficient volume resuscitation. All the patients adhered to the standard treatment protocol of the Surviving Sepsis Campaign guidelines [19].

### 2.2. Data Collection

Patient data, including age, sex, initial vital signs, quick SOFA, Glasgow Coma Scale (GCS), and Charlson Comorbidity Index (CCI) scores [20], were collected for demographic analysis. Regular laboratory blood tests were performed during enrollment. Sources of infection were documented through cultures of blood, tracheal aspiration, sputum, urine, body fluid, and pus. All bacteria were subjected to the minimum inhibitory concentration tests by using the VITEK^®^2 automated system (bioMérieux, Lyon, France). Antibiotic resistance was manually examined using the BBL Sensi-Disc test or automatically determined by the VITEK^®^2 automated system in our hospital. Additional tests for pneumonia pathogens included urine antigens of *Legionella pneumoniae* and *Streptococcus pneumoniae,* and immunoglobulin M of *Mycoplasma pneumoniae* and *Chlamydia pneumoniae*. In patients with sepsis, for the purpose of rapidly tracing multi-drug-resistant organisms, a film array (BioFire Diagnostics, Salt Lake City, UT, USA) was used as a pneumonia panel for specimens from tracheal aspiration and blood culture identification of bacterial growth in the blood culture panel at 48 h without definite culture results. Clinical outcomes, including the length of ICU stay, shock episodes within 48 h of ICU admission, hospitalization duration, and survival status, were recorded.

### 2.3. Free Hemoglobin, Cathepsin D, Cathepsin G, LVV-H7, and Angiotensin II Enzyme-Linked Immunosorbent Assay (ELISA) Analysis

Blood specimens from both ICU and healthy controls were stored at −80 °C until analysis. The angiotensin II ELISA (ADI-900-204; Enzo Life Sciences Inc., Farmingdale, NY, USA) was used, and all procedures were conducted according to the manufacturer’s instructions. For Hb metabolism, LVV-H7 was selected as the final stable form. Subsequently, cathepsin D (ab119586; Abcam, Cambridge, UK), cathepsin G (EC3237-1, Assaypro, St. Charles, MO, USA), Hb (ab157707; Abcam, Cambridge, UK), and LVV-H7 (MBS8820153; MyBioSource, San Diego, CA, USA) antibodies were added to the wells. After incubation, the washing step was repeated, and streptavidin horseradish peroxidase solution was added. The plate was washed again, tetramethylbenzidine substrate was added, and the plate was incubated in the dark. A stop solution was used to terminate the reaction, and 450 nm was chosen as the reference wavelength to measure absorbance. All tests were performed in duplicate.

### 2.4. Statistical Analysis

Categorical data are presented as frequencies and percentages, and the continuous data are presented as mean ± standard deviation. A one-way analysis of variance was used to compare among the three groups. The two continuous variables were compared using Student’s *t*-test, and categorical variables were compared using the chi-square test. Multivariate logistic regression analysis was performed, and confounding factors were adjusted using stepwise forward enrollment methods to identify Hb metabolite biomarkers for predicting sepsis, shock, and septic shock. Statistical analyses were performed using the IBM SPSS Statistics statistical software for Windows (version 26.0; IBM Corp, Armonk, NY, USA); statistical significance was set at *p* < 0.05.

## 3. Results

### 3.1. Hb Metabolic Biomarkers Among Sepsis, Non-Sepsis, and Healthy Control Groups

**Figure 2:**
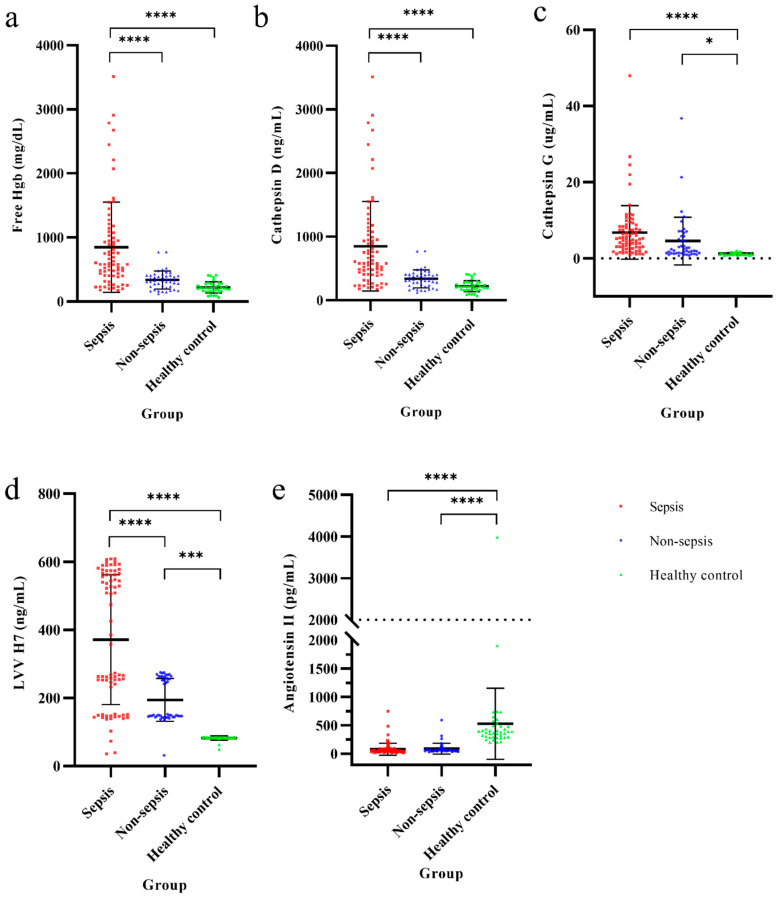
Hemoglobin metabolism among sepsis, non-sepsis, and health controls. (**a**) CFH, (**b**) cathepsin D, (**c**) cathepsin G, (**d**) LVV-H7 and (**e**) angiotensin II. CFH: Cell-free hemoglobin; LVV-H7: LVV-hemorphin-7. *p*-values calculated using Student’s *t*-test are shown above the scatter points. * *p* < 0.05, *** *p* < 0.001, **** *p* < 0.0001 for the difference between paired scatter points, respectively.

In total, 329 critically ill patients met the inclusion criteria of acute SOFA score change ≥ 2. After applying the exclusion/inclusion criterion, 120 patients were enrolled in the study (Figure 1), with 75 in the sepsis group and 45 in the non-sepsis groups. The control group consisted of blood samples from 40 healthy individuals. Post hoc analyses revealed that CFH levels were significantly higher (*p* < 0.001) in the sepsis group (15.3 ± 8.8 mg/dL) compared to the non-sepsis (9.8 ± 3.5 mg/dL) and control (9.3 ± 3.9 mg/dL) groups as Figure 2. Similarly, cathepsin D and LVV-H7 levels were significantly higher in the sepsis group (849 ± 703.1 and 371.7 ± 190.5 ng/mL, respectively) (*p* < 0.001) than in the non-sepsis (337.4 ± 141.1 and 194.5 ± 63.1 ng/mL, respectively) and control (220.4 ± 86.5 and 82.3 ± 6.4 ng/mL, respectively) groups. Cathepsin G levels were significantly higher (*p* < 0.001) in the sepsis (6.8 ± 7.0 µg/mL) and non-sepsis (4.5 ± 6.3 µg/mL) groups than in the control group (1.2 ± 0.3 µg/dL). However, angiotensin II levels were significantly lower in the sepsis (78.5 ± 105.8 ng/mL) and non-sepsis (87.8 ± 95.5 ng/mL) groups compared to the control group (527.1 ± 625.6 ng/mL). Significant differences in free Hb, cathepsin D, cathepsin G, LVV-H7, and angiotensin II values were observed between critically ill patients with acute changes in SOFA scores and the control group.

### 3.2. The Isolated Microorganisms in the Sepsis Group and the Diagnoses in the Non-Sepsis Group

**Figure 3:**
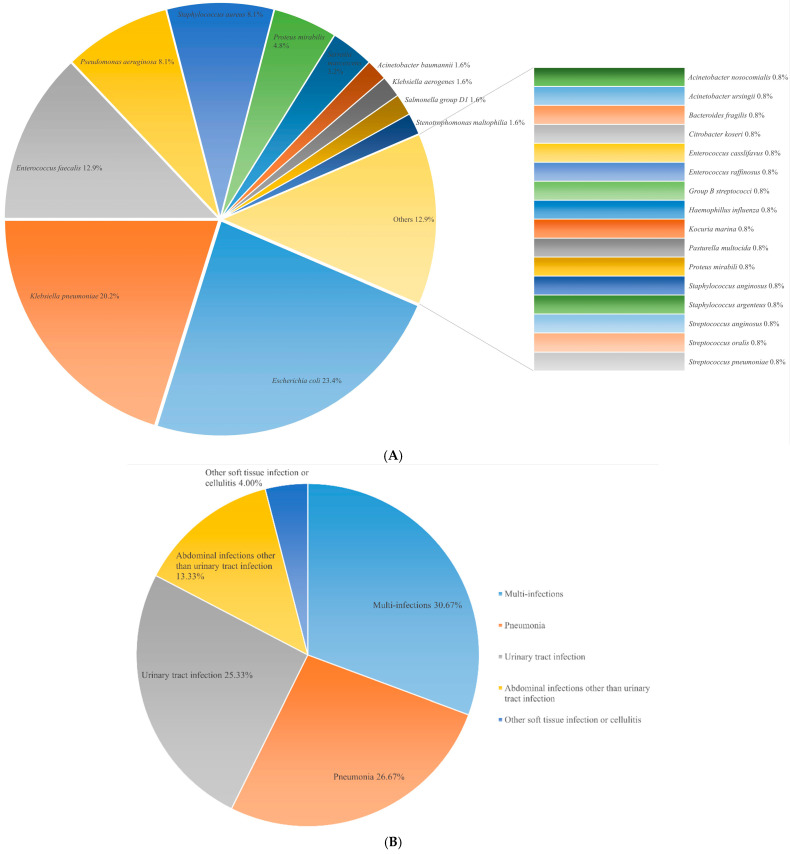

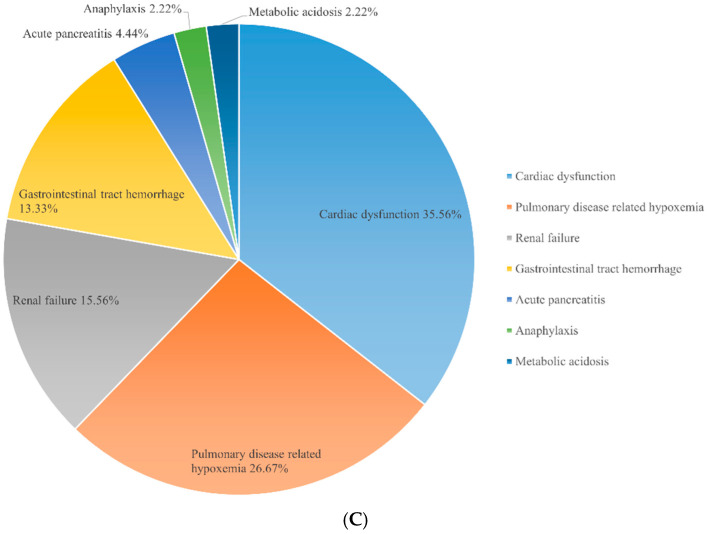
Etiologies of organ dysfunction in sepsis and non-sepsis groups. (**A**) Distribution of pathogens in the sepsis group; (**B**) Primary sources of infection in the sepsis group; (**C**) Major causes of multiple organ dysfunction in the non-sepsis group. The distribution of pathogens in the sepsis group was as follows: *Escherichia coli* (n = 29, 23.4%), *Klebsiella pneumoniae* (n = 25, 20.2%), *Enterococcus faecalis* (n = 16, 12.9%), *Pseudomonas aeruginosa* (n = 10, 8.1%), *Staphylococcus aureus* (n = 10, 8.1%), *Proteus mirabilis* (n = 6, 4.8%), *Serratia marcescens* (n = 4, 3.2%), and others (n = 24, 19.3%) (Figure 3). The primary sources of infection in the sepsis group included pneumonia (n = 20, 26.7%), urinary tract infection (n = 19, 25.3%), abdominal infections other than urinary tract infection (n = 10, 13.3%), other soft tissue infections or cellulitis (n = 3, 4.0%), and multi-infections (n = 23, 30.7%). The major causes of multiple organ dysfunction in the non-sepsis group were as follows: cardiac dysfunction (n = 16, 35.6%), pulmonary disease-related hypoxemia (n = 12, 26.7%), renal failure (n = 7, 15.6%), gastrointestinal tract hemorrhage (n = 6, 13.3%), acute pancreatitis (n = 2, 4.4%), metabolic acidosis (n = 1, 2.2%), and anaphylactic hypotension (n = 1, 2.2%).

The distribution of pathogens and the primary sources of infection in the sepsis (definite and probable) group, along with the diagnoses contributing to organ dysfunction in the non-sepsis (possible sepsis) group, are presented in Figure 3.

### 3.3. Comparisons of Parameters Between Sepsis and Non-Sepsis Groups

Table 1 shows the demographic characteristics of the sepsis and non-sepsis groups. Significant differences were observed in initial body temperature (BT; 36.99 vs. 36.38 °C, *p* < 0.05), MAP (82.5 vs. 97.6 mmHg, *p <* 0.001), and SOFA score (6.6 vs. 5.1, *p* < 0.05) between the sepsis and non-sepsis groups. Clinical variables and outcomes were similar between the two groups, except for shock episodes, which were significantly higher in the sepsis group (81.3% vs. 44.4%, *p <* 0.001).

There were no significant differences between the two groups in red blood cell count, Hb, platelets, monocytes, sodium (Na), potassium (K), alanine transaminase, aspartate transaminase, albumin, blood urea nitrogen, creatinine, random glucose, lactate, prothrombin time, international normalized ratio, pH, SaO_2_ in arterial blood gas, ratio of arterial oxygen partial pressure to fractional inspired oxygen, cathepsin G, and angiotensin II levels (Table 2). However, significantly higher levels of white blood cell (WBC) count (13.16 vs. 9.36 × 10^3^/µL, *p* < 0.001), total bilirubin (1.71 vs. 0.95 mg/dL, *p* < 0.05), activated partial thromboplastin time (aPTT) (32.65 vs. 28.61 s, *p* < 0.05) levels, neutrophil (10.12 vs. 6.91 × 10^3^/µL, *p* < 0.001), C-reactive protein (CRP) (13.50 vs. 5.11 mg/dL, *p <* 0.001) were observed in the sepsis group than in the non-sepsis group, respectively. In contrast, lower lymphocyte levels (0.91 vs. 1.71 × 10^3^/µL, *p* < 0.05) were detected in the sepsis group.

As shown in Figure 2 and Table 2, the sepsis group had significantly higher levels of free Hb (15.27 vs. 9.75 mg/dL; *p <* 0.001), cathepsin D (849.19 vs. 337.44 ng/mL; *p* < 0.001), and LVV-H7 (371.68 vs. 194.48 ng/mL; *p* < 0.001) than the non-sepsis group. However, there were no significant differences observed in cathepsin G or angiotensin II levels between the two groups.

### 3.4. Potential Factors for Diagnosis of Sepsis, Shock, or Septic Shock in Critical Ill Patients with Acute Change of SOFA Score ≥ 2

Forward stepwise logistic regression identified variables with *p* < 0.05 as significant factors associated with sepsis classifications. Significant independent variables were CRP (odds ratio [OR] 1.086, 95% confidence interval [CI] 1.024–1.153; *p* < 0.001), aPTT (OR 0.999, 95% CI 0.999–1.163; *p* < 0.05), and WBC (OR 1.128, 95% CI 1.017–1.251; *p* < 0.05). After adjusting for other factors, LVV-H7 (adjusted OR [aOR] 1.009, 95% CI 1.005–1.013; *p <* 0.001) was a significant factor for differentiating sepsis classifications (Table 3).

**Figure 4:**
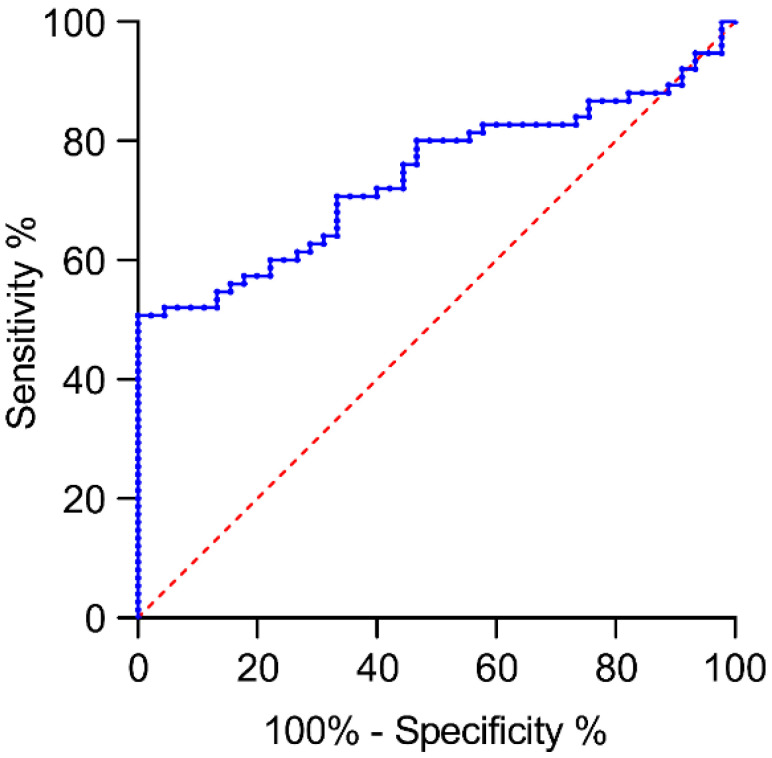
The receiver operating characteristic (ROC) curve for LVV-H7 in predicting sepsis. The blue solid line represents the ROC curve of LVV-H7 (LVV-hemorphin-7), illustrating its diagnostic performance. The red dashed line indicates the 50% area under the curve (AUC), serving as the reference line for random chance.

The receiver operating characteristic (ROC) curves were calculated to predict sepsis using different biomarkers (Table 4 and Figure 4). The area under the curve (AUC) was 0.741 for LVV-H7, with the best cutoff point being 316.61 ng/mL (50.7% sensitivity and 100.0% specificity). The AUC was 0.738 for CRP, and a cutoff point of 5.85 mg/dL (72% sensitivity and 77.8% specificity. The AUC was 0.639 in WBC, with a cutoff point of 12.03 × 10^3^/µL (53.3% sensitivity and 82.2% specificity). According to Youden’s J statistic and AUC, LVV-H7 was the best biomarker for the diagnosing of sepsis, with high specificity and low sensitivity (Figure 4).

Forward stepwise logistic regression identified significant independent variables with *p* < 0.05 for predicting shock or septic shock. Among the 120 ICU patients, 81 experienced shock episodes within 48 h of ICU admission. Significant independent variables for predicting shock were Cathepsin G (aOR 0.917, 95% CI 0.848–0.991; *p* < 0.05), LVV-H7 (aOR 1.005, 95% CI 1.002–1.008; *p* < 0.05), SOFA score (OR 1.341, 95% CI 1.103–1.630; *p* < 0.05), and aPTT (OR 1.096, 95% CI 1.015–1.184; *p* < 0.05).

For predicting septic shock, among the 120 ICU patients, 40 were identified within 48 h of ICU admission. Significant independent variables for predicting septic shock were cathepsin D (aOR 1.001, 95% CI 1.000–1.002; *p* < 0.05), lactate (OR 1.182, 95% CI 1.054–1.324; *p* < 0.05), and CRP (OR 1.094, 95% CI 1.044–1.147; *p* < 0.001) (Table 4).

## 4. Discussion

This study is the first to evaluate Hb metabolism biomarkers, including free Hb, cathepsin D, cathepsin G, LVV-H7, and angiotensin II, in relation to sepsis classifications among critically ill patients with an acute increase in SOFA score ≥ 2.

**Figure 5:**
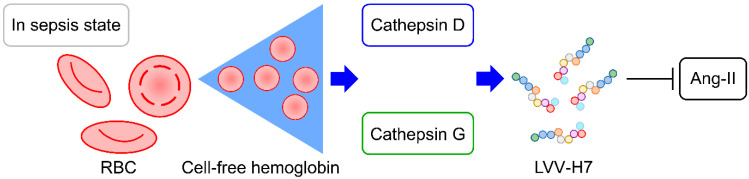
The signal transduction mechanism during sepsis. In sepsis state, RBC release cell-free hemoglobin due to infection, cathepsin D and cathepsin G degrade CFH, thereby releasing opioid peptides LVV-H7. The LVV-H7 binding to ANG-II, causing blood pressure decrease in patients with sepsis. CFH: cell-free hemoglobin; LVV-H7: LVV-hemorphin-7; RBC: red blood cells.

Our findings demonstrate that critically ill definite and probable sepsis patients had higher levels of CFH, cathepsin G, cathepsin D, and LVV-H7 compared to possible sepsis patients and healthy controls, indicating potential metabolic interactions between immune cell lysosome enzymes and CFH during sepsis (Figure 5). These results align with previous research suggesting that CFH contributes to oxidative and endothelial injury in sepsis-related acute respiratory distress syndrome [21], a process well-documented in hemolysis and CFH release [4]. The CFH released into the blood increases the chances of cathepsin G originating from neutrophils and cathepsin D originating from macrophages digesting free Hb. Although cathepsin G levels were higher in the definite and probable sepsis group, it was not significantly different from those in the possible sepsis group, possibly because cathepsin G is released by neutrophils during inflammation and contributes to the immune response when the body experiences infection or inflammation [22,23]. Similarly, although cathepsin D levels were significantly higher in the definite and probable sepsis patients, they did not show a positive association in the logistic regression model for sepsis classification, possibly because cathepsin D is released from macrophages in severe acidic environments, leading to variable blood concentrations that may not accurately reflect sepsis status [24]. Despite the lack of association for cathepsins G and D in predicting sepsis classifications, these enzymes contribute to the production of downstream metabolites, such as LVV-H7, which was identified as a stable and predominant blood biomarker [12,25]. In this study, LVV-H7 was a significant factor for sepsis classifications (Table 3), although its levels may be influenced by comorbidities like cancer [26], obesity [27], and diabetes [28]. Here, malignancies, diabetes, and BMI calculations were investigated, and no differences in their distributions between the different sepsis groups were found. Notably, LVV-H7 demonstrated high specificity (100%) but low sensitivity (50.7%) for sepsis classifications, suggesting it may be more useful as an exclusionary marker rather than a primary diagnostic tool, similar to the role of D-dimer in ruling out pulmonary embolism [29].

WBC was the traditional biomarker of systemic inflammation response syndrome as a previous diagnostic criterion of sepsis but was changed to the SOFA score at the 3rd International Sepsis Conference [18]. Here, WBC count was still a strong predictive factor for sepsis. In addition, the CRP level was also a traditional prognostic factor for sepsis in a clinical study [30] and was not included in any disease severity scoring system. Thus, the CRP level remained a substantial predictive factor for sepsis in this study.

In terms of clinical outcomes, the definite and probable sepsis group experienced significantly more shock episodes than the possible sepsis group, although no difference in the length of ICU stay, hospital stay, and survival. Our analysis of Hb metabolites (LVV-H7, cathepsin D, cathepsin G, and angiotensin II) in relation to shock and septic shock revealed that LVV-H7 could predict shock, while cathepsin G showed potential in reducing shock episodes (Table 5). The relationship between LVV-H7 and angiotensin II levels suggested dual mechanisms, with LVV-H7 potentially influencing blood pressure regulation through both angiotensin-converting enzyme inhibition and direct targeting of the angiotensin II type 1 receptor (AT1R) [14]. These mechanisms result in hypotension driven by LVV-H7, rather than by angiotensin II. Conversely, cathepsin G showed potential to predict less shock in multivariate logistic regression (Table 5). Cathepsin G traditionally acts as neutrophil angiotensin II-generating protease with functional control of blood pressure [31]. Cathepsin G may contribute to angiotensin II production in pig renal tissue [32], and these mechanisms may explain our clinical results that cathepsin G regulates the blood pressure with fewer shock episodes. Furthermore, cathepsin D showed potential to predict septic shock (Table 5). Septic shock has strict criteria of profound shock with lactic acidosis. Previous studies showed that cathepsin D is released more in acidic environments [24], suggesting an association with septic shock with lactic acidosis. Therefore, the lysosomal enzyme (cathepsin G and cathepsin D), CFH metabolites (LVV-H7), and angiotensin II may act as different indicators to guide clinical infection outcomes, including sepsis, shock, or septic shock.

Procalcitonin is a highly sensitive diagnostic marker for sepsis and is also used to monitor the effectiveness of antibiotic therapy [33]. However, in our study, PCT was not associated with sepsis classifications. The possible reasons are as follows. Our study was designed to differentiate the causes of systemic inflammatory response syndrome (multiple organ and tissue hypoperfusion) and the hemoglobin metabolism status between definite, probable, and possible sepsis. Since all patients met the sepsis criteria, the possible sepsis group still exhibited high PCT levels. Another reason could be that we excluded cases where clinical cultures were collected 48 h after ICU admission. This exclusion might have affected the possible sepsis group, as some patients may have had insidious bacterial growth leading to high PCT levels. The exclusion was intended to prevent secondary infections from confounding the study results.

Some limitations of this study include the small sample size of the non-sepsis group. Although disease severity and comorbidities were similar between the sepsis and non-sepsis groups, the sample size in the non-sepsis and control groups, which was 50% smaller than the sepsis group, may have reduced the statistical power and impacted the results of LVV-H7 as a less sensitive biomarker. Additionally, the SOFA score, a major diagnostic criterion for sepsis (acute change > 2), made it challenging for the non-sepsis and control groups to meet the inclusion criteria, resulting in exclusions based on these criteria. Increasing the control group size may enhance the statistical power for case–control study comparisons. Future studies should consider multicenter prospective designs enrolling sepsis and non-sepsis cases in a 1:1 ratio with SOFA > 2 as control groups. Secondly, in the sepsis group (n = 75), the diversity of organisms involved in sepsis may have affected the validation of sepsis biomarkers. Future comparisons between different classifications, such as Gram-positive vs. Gram-negative, multidrug-resistant organisms vs. non-resistant, and bacterial vs. viral infections, could help clarify the characteristics of new sepsis biomarkers. Additionally, we did not test procalcitonin more than once in the non-sepsis group for a series of comparisons. Procalcitonin is more sensitive to bacterial infection and suitable for new biomarker comparisons. We suggest routine procalcitonin testing as a follow-up measure in future sepsis studies.

## 5. Conclusions

LVV-H7, cathepsin D, and cathepsin G levels serves as potential biomarkers for sepsis classifications and shock episodes in critically ill patients with acute changes in the SOFA score ≥ 2. LVV-H7 may help exclude definite and probable sepsis when levels are below normal. Cathepsin G may assist in blood pressure control and reduce shock incidence, while cathepsin D is more active and associated with septic shock. Further multicenter prospective studies that enroll other non-sepsis control groups are needed to validate the clinical utility of cathepsin D, cathepsin G, and LVV-H7 in differentiating sepsis and septic shock.

## Figures and Tables

**Table 1:** Characteristics of the different sepsis groups of the study population (n = 120).

Variables	Definite and Probable Sepsis (n = 75)	Possible and Non-Sepsis (n = 45)	*p*-Value
Age (years)	68.81 ± 14.17	71.09 ± 13.56	0.388 ^c^
Sex			0.530 ^a^
Female	31 (41.3%)	16 (35.6%)	
Male	44 (58.7%)	29 (64.4%)	
Body mass index (kg/m^2^)	22.96 ± 4.73	22.85 ± 4.41	0.900 ^c^
Vital signs			
Body temperature (°C)	36.99 ± 1.45	36.38 ± 0.81	0.004 ^c^*
Respiratory rate (/min)	21.17 ± 5.38	20.76 ± 4.69	0.667 ^c^
Heart rate (/min)	106.33 ± 25.76	100.16 ± 26.57	0.211 ^c^
MAP (mmHg)	82.53 ± 22.00	97.58 ± 21.33	<0.001 ^c^**
SpO_2_ (%)	94.85 ± 4.94	94.36 ± 5.03	0.597 ^c^
GCS	12.13 ± 4.06	12.27 ± 4.08	0.862 ^c^
CCI	5.55 ± 2.80	5.38 ± 2.31	0.734 ^c^
Comorbidities			
Diabetes	33 (44.0%)	18 (40.0%)	0.668 ^a^
Cardiovascular disease	26 (34.7%)	22 (48.9%)	0.124^a^
Chronic kidney disease	24 (32.0%)	12 (26.7%)	0.537 ^a^
Neurologic diseases	19 (25.3%)	11 (24.4%)	0.913 ^a^
Pulmonary disease	15 (20.0%)	12 (26.7%)	0.397 ^a^
Malignancy	15 (20.0%)	6 (13.3%)	0.352 ^a^
Chronic liver disease	8 (10.7%)	4 (8.9%)	1.000 ^b^
Autoimmune disease	2 (2.7%)	2 (4.4%)	0.630 ^b^
Disease severity			
qSOFA	1.09 ± 0.83	0.84 ± 0.74	0.099 ^c^
SOFA score	6.61 ± 2.91	5.11 ± 2.80	0.006 ^c^
APACHE II score	23.07 ± 9.92	21.36 ± 8.40	0.336 ^c^
Oxygenation device status			0.512 ^a^
IMV	35 (46.7%)	26 (57.8%)	
NIV	5 (6.7%)	4 (8.9%)	
Oxygen supplement	31 (41.3%)	14 (31.1%)	
Oxygen not needed	4 (5.3%)	1 (2.2%)	
Clinical outcomes			
Length of stay in ICU	12.44 ± 12.84	15.31 ± 14.65	0.263 ^c^
Hospital days	23.20 ± 19.71	27.36 ± 23.91	0.307 ^c^
Shock episodes	61 (81.3%)	20 (44.4%)	<0.001 ^a^**
Survival	54 (72.0%)	35 (79.5%)	0.360 ^a^

^a^ A chi-square test is used for the comparison of categorical variables, ^b^ Fisher’s exact test is used for the comparison of categorical variables, ^c^ an independent t-test is used for continuous variables between the sepsis and non-sepsis groups. * *p* < 0.05; ** *p* < 0.001. APACHE, Acute Physiology and Chronic Health Evaluation; CCI, Charlson Comorbidity Index; GCS, Glasgow Coma Scale; MAP, mean arterial pressure; SOFA, Sequential Organ Failure Assessment; SpO_2_, saturation from pulse oximeter; qSOFA, Quick Sequential Organ Failure Assessment; IMV, Invasive Mechanical Ventilation; NIV, Noninvasive Ventilation.

**Table 2:** Laboratory data from different groups of the study population (n = 120).

Variables	Definite and Probable Sepsis (n = 75)	Possible and Non-Sepsis (n = 45)	*p*-Value
WBC (10^3^/µL)	13.16 ± 7.71	9.36 ± 4.27	0.001 *
RBC (10^6^/µL)	3.67 ± 0.84	3.60 ± 0.92	0.650
Hemoglobin (g/dL)	11.00 ± 2.58	10.97 ± 2.58	0.948
Platelets (10^3^/µL)	186.84 ± 129.37	202.96 ± 93.94	0.433
Neutrophil (10^3^/µL)	10.12 ± 6.70	6.91 ± 3.64	0.001 *
Lymphocyte (10^3^/µL)	0.91 ± 0.80	1.71 ± 1.96	0.011 *
Monocyte (10^3^/µL)	0.50 ± 0.41	0.47 ± 0.31	0.602
Na (mEq/L)	135.13 ± 6.93	136.76 ± 5.22	0.149
K (mEq/L)	4.23 ± 1.04	4.17 ± 0.84	0.748
AST (U/L)	92.60 ± 167.37	71.24 ± 147.65	0.481
ALT (U/L)	41.43 ± 59.41	39.49 ± 42.64	0.849
Albumin (g/dL)	3.00 ± 0.58	3.19 ± 0.49	0.075
BUN (mg/dL)	52.77 ± 41.87	43.53 ± 32.13	0.206
Creatinine (mg/dL)	2.93 ± 2.57	2.68 ± 3.19	0.642
Random Glucose (mg/dL)	179.30 ± 80.1	176.15 ± 82.68	0.837
Lactate (mmol/L)	3.62 ± 3.49	3.75 ± 4.57	0.863
CRP (mg/dL)	13.50 ± 11.38	5.11 ± 7.53	<0.001 **
PCT (ng/mL)	19.03 ± 37.18	14.57 ± 43.85	0.553
Total bilirubin (mg/dL)	1.71 ± 2.56	0.95 ± 0.77	0.019 *
PT (second)	12.30 ± 2.49	11.55 ± 1.84	0.084
INR (ratio)	1.23 ± 0.32	1.18 ± 0.32	0.404
aPTT (second)	32.65 ± 8.04	28.61 ± 6.12	0.002 *
ABG pH	7.36 ± 0.12	7.39 ± 0.10	0.093
ABG SaO_2_	95.89 ± 3.43	96.82 ± 3.43	0.154
P/F ratio	243.97 ± 137.03	289.95 ± 148.78	0.088
Hemoglobin catabolism			
Free Hemoglobin (mg/dL)	15.27 ± 8.81	9.75 ± 3.51	<0.001 **
Cathepsin D (ng/mL)	849.19 ± 703.08	337.44 ± 141.09	<0.001 **
Cathepsin G (μg/mL)	6.79 ± 7.04	4.54 ± 6.28	0.080
LVV-H7 (ng/mL)	371.68 ± 190.52	194.48 ± 63.10	<0.001 **
Angiotensin II (pg/mL)	78.50 ± 105.78	87.81 ± 95.54	0.630

An independent *t*-test was used to compare continuous variables between the sepsis and non-sepsis groups. * *p* < 0.05; ** *p* < 0.001. ABG, arterial blood gas; ALT, alanine transaminase; aPTT, activated partial thromboplastin time; AST, aspartate transaminase; BUN, blood urea nitrogen; CRP, C-reactive protein; INR, international normalized ratio; LVV-H7, LVV-hemorphin 7; PCT, procalcitonin; P/F ratio, ratio of arterial oxygen partial pressure to fractional inspired oxygen; PT, prothrombin time; RBC, red blood cell count; SaO_2_, arterial oxygen saturation; WBC, white blood cell.

**Table 3:** Logistic regression models of sepsis classification factors.

Variables	β	SE	OR	95% CI		*p*-Value
				Lower	Upper	
LVV-H7	0.009	0.002	1.009	1.005	1.014	<0.001 **
CRP	0.083	0.030	1.086	1.024	1.153	0.006 *
aPTT	0.075	0.039	1.078	0.999	1.163	0.052
WBC	0.121	0.053	1.128	1.017	1.251	0.022 *

* *p* < 0.05; ** *p* < 0.001. Logistic regression model with forward stepwise selection for variables such as free hemoglobin, cathepsin D, cathepsin G, LVV-H7, angiotensin II, SOFA score, initial body temperature, MAP, Total bilirubin, CRP, aPTT, WBC, neutrophils, and lymphocytes. aPTT, activated partial thromboplastin time; CI, confidence interval; CRP, C-reactive protein; LVV-H7, LVV-hemorphin 7; OR, odds ratio; SE, standard error; WBC, white blood cell.

**Table 4:** ROC curves of different biomarkers of sepsis classifications (n = 120).

Biomarkers	Area	95% CI		*p*-Value	Cuff Point	Sensitivity	Specificity	Youden’s J
		Lower	Upper					
LVV-H7	0.741	0.654	0.827	<0.001 **	316.61 (ng/mL)	0.507	1.000	0.507
CRP	0.738	0.647	0.830	<0.001 **	5.85 (mg/dL)	0.720	0.778	0.498
WBC	0.639	0.540	0.738	0.011 *	12.03 (10^3^/µL)	0.533	0.822	0.356

Notes: Youden’s J statistic: sensitivity + specificity −1. * *p* < 0.05; ** *p* < 0.001. CI, confidence interval; CRP, C-reactive protein; LVV-H7, LVV-hemorphin 7; ROC, receiver operating characteristic; WBC, white blood cell.

**Table 5:** Logistic regression models of shock or septic shock factors.

Variables	β	SE	OR	95% CI		*p*-Value
				Lower	Upper	
For shock factors						
Cathepsin G	−0.087	0.040	0.917	0.848	0.991	0.030 *
LVV-H7	0.005	0.002	1.005	1.002	1.008	0.004 *
SOFA score	0.293	0.100	1.341	1.103	1.630	0.003 *
aPTT	0.092	0.039	1.096	1.015	1.184	0.019 *
For septic shock factors						
Cathepsin D	0.001	0.000	1.001	1.000	1.002	0.026 *
Lactate	0.167	0.058	1.182	1.054	1.324	0.004 *
CRP	0.090	0.024	1.094	1.044	1.147	<0.001 **

* *p* < 0.05; ** *p* < 0.001. Logistic regression model with forward stepwise selection for variables such as free hemoglobin, cathepsin D, cathepsin G, LVV-H7, Angiotensin II, SOFA score, gender, BMI, CCI, APACHE II score, lactate, oxygenation device status, albumin, CRP, PT, aPTT, INR, WBC, neutrophils, and lymphocytes. APACHE, Acute Physiology and Chronic Health Evaluation; aPTT, activated partial thromboplastin time; BMI, body mass index; CCI, Charlson comorbidity index; CI, confidence interval; CRP, C-reactive protein; INR, international normalized ratio; LVV-H7, LVV-hemorphin 7; OR, odds ratio; PT, prothrombin time, SE, standard error; SOFA, Sequential Organ Failure Assessment; WBC, white blood cell.

## Data Availability

The data supporting the findings of this study are available from the Taipei Tzu Chi Hospital, Buddhist Tzu Chi Medical Foundation. Restrictions may apply to the availability of data used under the license of the current study because they are not publicly available. However, the data are available from the authors upon reasonable request and with permission from Taipei Tzu Chi Hospital and the Buddhist Tzu Chi Medical Foundation.

## List of Abbreviations

aHR, adjusted hazard ratio; aOR, adjusted odds ratio; aPTT, activated partial thromboplastin clotting time; AUC: area under the curve; BMI, body mass index; BT: body temperature; BUN, blood urea nitrogen; CCI, Charlson comorbidity index; CI, confidence interval; CFH: cell-free hemoglobin; CRP, C-reactive protein; ELISA, enzyme-linked immunosorbent assay; GCS, Glasgow Coma Scale; Hb, hemoglobin; LVV-H7, LVV-hemorphin-7; MAP, Mean arterial pressure; PCT, procalcitonin; qSOFA, quick sepsis-related organ failure assessment; RBC: red blood cells; SOFA, Sequential Organ Failure Assessment Score; SpO_2_, oxygen saturation of pulse oximeter; WBC: white blood cells.
